# Walking in a heterogeneous landscape: Dispersal, gene flow and conservation implications for the giant panda in the Qinling Mountains

**DOI:** 10.1111/eva.12686

**Published:** 2018-08-31

**Authors:** Tianxiao Ma, Yibo Hu, Isa‐Rita M. Russo, Yonggang Nie, Tianyou Yang, Lijuan Xiong, Shuai Ma, Tao Meng, Han Han, Ximing Zhang, Michael W. Bruford, Fuwen Wei

**Affiliations:** ^1^ Key Laboratory of Animal Ecology and Conservation Biology Institute of Zoology Chinese Academy of Sciences Beijing China; ^2^ University of Chinese Academy of Sciences Beijing China; ^3^ Center for Excellence in Animal Evolution and Genetics Chinese Academy of Sciences Kunming China; ^4^ Cardiff School of Biosciences Cardiff University Cardiff UK; ^5^ School of Life Sciences Guizhou Normal University Guiyang Guizhou China; ^6^ Guangxi Forest Inventory & Planning Institute Nanning Guangxi China; ^7^ Changqing National Nature Reserve Shaanxi China; ^8^ Sustainable Places Research Institute Cardiff University Cardiff UK

**Keywords:** isolation‐by‐barriers, isolation‐by‐distance, isolation‐by‐resistance, landscape genetics, topographic variables

## Abstract

Understanding the interaction between life history, demography and population genetics in threatened species is critical for the conservations of viable populations. In the context of habitat loss and fragmentation, identifying the factors that underpin the structuring of genetic variation within populations can allow conservationists to evaluate habitat quality and connectivity and help to design dispersal corridors effectively. In this study, we carried out a detailed, fine‐scale landscape genetic investigation of a giant panda population from the Qinling Mountains for the first time. With a large microsatellite data set and complementary analysis methods, we examined the role of isolation‐by‐barriers (IBB), isolation‐by‐distance (IBD) and isolation‐by‐resistance (IBR) in shaping the pattern of genetic variation in this giant panda population. We found that the Qinling population comprises one continuous genetic cluster, and among the landscape hypotheses tested, gene flow was found to be correlated with resistance gradients for two topographic factors, slope aspect and topographic complexity, rather than geographical distance or barriers. Gene flow was inferred to be facilitated by easterly slope aspect and to be constrained by topographically complex landscapes. These factors are related to benign microclimatic conditions for both the pandas and the food resources they rely on and more accessible topographic conditions for movement, respectively. We identified optimal corridors based on these results, aiming to promote gene flow between human‐induced habitat fragments. These findings provide insight into the permeability and affinities of giant panda habitats and offer important reference for the conservation of the giant panda and its habitat.

## 1 INTRODUCTION

As key population parameters, individual dispersal, gene flow and their demographic consequences can profoundly affect population dynamics and have important ecological and evolutionary consequences. Gene flow is one mechanism that can maintain population genetic diversity and increase the likelihood of evolutionary adaptation, while demographic isolation will induce population genetic structuring and even lead to reproductive isolation and eventually speciation (Slatkin, 1987). Several factors can motive the dispersal of individuals, including avoidance of inbreeding (Waser, Austad, & Keane, 1986) and competition (including among kin, Hamilton & May, 1977), and spatial heterogeneity within habitats can act synergistically with these and other factors (Storfer, Murphy, Spear, Holderegger, & Waits, 2010).

For species with moderate dispersal, an isolation‐by‐distance (IBD) model has been classically invoked to describe the relationship between geographic and genetic variation. It predicts lower genetic similarity between individuals with increasing geographical distance, as the homogenizing effects of gene flow diminish across space (Hutchison & Templeton, 1999; Wright, 1943). However, the shape of this relationship may be complex. Many empirical studies have demonstrated that dispersal can be impeded by significant biotic (e.g., behavioural) and/or abiotic (e.g., physical) barriers (Hollatz et al., 2011; Vallinoto et al., 2006). These boundaries may result in fine‐scale genetic structure within populations and which is consistent with the hypothesis of isolation‐by‐barriers (IBB). Habitat heterogeneity, via landscape configuration and other environmental effects, can also strongly affect gene flow by promoting or hampering individual movements (Cushman, McKelvey, Hayden, & Schwartz, 2006; McRae, 2006). The magnitude of the impact of landscape features on dispersal can also be described in terms of resistance, and isolation‐by‐resistance (IBR) models have been developed to evaluate the correlation between landscape composition and genetic differentiation (McRae, 2006).

In natural populations, the factors described above may not work alone in shaping patterns of genetic variation. Thus, a simple null‐hypothesis test may elicit undetected or misinterpreted correlated signals, which would result in spurious inference on the role of particular factors in genetic differentiation or result in omission of authentic signals. Such misinterpretation could compromise future management and conservation of populations involving, for example, in situ habitat restoration and the configuration of dispersal corridors. Against this background, a comprehensive analytical framework is required, which simultaneously investigates the effects of IBD, IBB and IBR (Cushman & Landguth, 2010; Cushman et al., 2006; Ruiz‐Gonzalez, Cushman, Madeira, Randi, & Gómez‐Moliner, 2015). This approach is of particular importance for natural populations of threatened species, which face complex spatial effects because of small population size (e.g., Allee effects), fragmented habitat and human‐induced disturbance. By jointly considering alternative hypotheses, more reliable conclusions on the key determinants of spatial genetic variation and dispersal processes can be drawn, even in the absence of direct observational data.

The emblematic giant panda (*Ailuropoda melanoleuca*) is endemic to China and restricted to the mountains on the eastern edge of the Qinghai–Tibetan Plateau and to the north of the Sichuan basin. The giant panda's cryptic behaviour has made it difficult to directly observe important life history processes, including dispersal behaviour, rendering approaches such as landscape genetics a promising solution to shed light on this process using an indirect method. To date, however, the sparse sampling that has been possible within and among the isolated regional populations of this species have resulted in only a few landscape genetic studies, including Zhan et al. (2007, 2007) and Hu, Zhan, Qi, and Wei (2010), who both detected a female‐biased dispersal pattern, and Zhu, Zhan, Meng, Zhang, and Wei (2010) who detected the strong barrier effect of large rivers on gene flow. However, the recently reported Fourth National Survey on Giant Pandas (State Forestry Administration 2015) involved intensive sampling efforts and habitat surveys and has provided an unusual opportunity to investigate genetic structure and its causal factors in giant panda populations.

Here, we focus on the genetically distinct giant panda population located in the southern slopes of the Qinling Mountains in central China. The Qinling population is distinguished genetically from other Sichuan populations (Lu et al., 2001; Zhang et al., 2013; Zhao et al., 2013). A recent population genomics study inferred that the Qinling and Sichuan populations diverged about 300,000 years ago (Zhao et al., 2013). Zhang et al. (2007) suggested that the Qinling population should be regarded as an independent management unit in conservation to conserve its locally adapted attributes and genetic integrity. However, information on the fine‐scale structure within this unique population is lacking. The giant panda's well‐known specialized diet makes its movement behaviour, including dispersal, largely dependent on the distribution of suitable bamboo forests. For example, giant pandas move seasonally between summer and winter habitats located at different elevations, following the availability of bamboo resources. Nevertheless, previous ecological studies have indicated that certain landscape features, such as steep slopes and forest configuration, could influence the dispersal of giant pandas at different scales (Qi, Hu, Gu, Li, & Wei, 2009; Qi et al., 2012; Zhang et al., 2011), but these observations lack a fine‐scale geospatial analysis that can be provided using landscape genetics. Furthermore, given the presence of severe habitat fragmentations, the key factors driving dispersal and shaping spatial pattern of genetic differentiation remain unclear for Qinling and indeed for all giant panda population (Wei, Zhang, & Hu, 2011; Wei et al., 2012; Wei, Hu et al., 2015; Wei, Swaisgood et al., 2015).

In consequence, our study aimed to examine the genetic status of the Qinling giant panda population and to address the following specific questions: (a) due to long isolation and substantial habitat fragmentation, does the Qinling population feature low genetic diversity compared to other giant panda populations; (b) as the Qinling population is subjected to considerable human activity, IBB, IBD, IBR or a combination of these models which play the main force in shaping the genetic differentiation pattern of this giant panda population. In answering these questions, conservationists can propose more reasonable and effective strategies in conserving the species and its habitats.

## 2 METHODS

### 2.1 Study area and sampling scheme

The Qinling Mountains, which are located in the south of Shaanxi province in central China, have been recognized as a natural boundary between north and south China, and it is also currently the northernmost distributional border of the giant panda. Different from the semi‐arid northern slopes of the area, the southern slopes feature a mild and moist habitat, providing giant pandas with a natural refugium (Pan et al., 2001). The Qinling giant panda population significantly differs from other giant panda populations, based on both morphological and genetic evidences (Chen et al., 2010; Wan, Fang, Wu, & Fujihara, 2003; Wan, Wu, & Fang, 2005; Zhao et al., 2013). However, in the Qinling Mountains, as elsewhere, suitable habitat for the giant panda has been declining since the beginning of recorded history in the region. By 2015, only 151,466 hectares (ha) of suitable habitat remained based on the Fourth National Survey of Giant Panda (State Forestry Administration 2015), mainly caused by anthropogenic activities in housing development, logging, road construction and agriculture.

A total of 537 faecal samples were collected from six counties located in the Qinling Mountains, including Foping, Yangxian, Zhouzhi, Taibai, Ningshan and Liuba. The samples were collected from March to May in 2012 using noninvasive genetic sampling method, following random transect lines at two scales. Based on the last large‐scale survey and recent occurrence records of giant pandas, the entire study area was classified into two types: core investigation areas and normal investigation areas. The core investigation areas were those defined as suitable habitat in previous surveys or using recent occurrence records of the animal. They were divided into a grid size of 2 km^2^ for a finer scale survey, while normal investigation areas were divided into a grid size of 6 km^2^. A transect with minimum length of 0.75 km was made in each survey grid. To collect more samples and cover different giant panda habitats, a zigzag movement path was adopted when collecting samples inside a survey grid, while a “U” shape or a circle movement path was adopted when crossing different grids.

### 2.2 Molecular analysis

We extracted total DNA from faecal samples using the Qiagen DNeasy Stool kit (QIAGEN^®^ Hilden, Germany) following the manufacturer's instructions. Twelve microsatellite loci, Ame‐μ10, μ11, μ13, μ15, μ22, μ24, μ26, μ27, AY79, AY95, AY217, and AY161213 (Lu et al., 2001; Shen et al., 2005; Wu et al. 2009, Supporting information Table S1), were amplified and genotyped for each sample. To obtain reliable results, a multitube approach (Taberlet et al., 1996) was applied. PCRs were conducted in a total volume of 10 μl, comprising 2 μl DNA, 5 μl HotStarTaq Master Mix (QIAGEN), 0.3 μM of the forward (5′ labelled with FAM, TAMRA or HEX) and reverse primers and 1 μg/μl BSA (Promega). PCRs were carried out in a Thermo MBS Thermal Cycler, starting with 94°C for 15 min, followed by a touchdown approach (a total of 35–39 cycles of 94°C/15 s, *T*_a_/30 s, 72°C/45 s) and a final step of 60°C for 30 min. *T*_a_ was decreased by 2°C every second cycle from 60°C to a touchdown temperature (48–50°C), which was used for an additional 25 cycles (Zhan et al., 2006). PCR products were separated using an ABI 3730xl sequencer and scored using GeneMarker^®^ v 2.2.0 (SoftGenetics LLC).

### 2.3 Genetic diversity analysis

The probability of pairs of individuals bearing an identical multilocus genotype (P(ID)) was calculated using GIMLET V1.3.1 (Valière, 2002) to confirm the discrimination power of the microsatellites used in this study. MICRO‐CHECKER (Van Oosterhout, Hutchinson, Wills, & Shipley, 2004) was applied to check for null alleles, large allele dropout or stuttering. Genetic diversity indices including the number of alleles per loci (*A*), the expected heterozygosities (H_E_) and the observed heterozygosities (H_O_) were computed using FASTAT 2.9.3.2 (Goudet, 2001), which was also used to test whether loci were deviated from Hardy–Weinberg equilibrium. Linkage disequilibrium between pairs of loci and across the whole data set was assessed using FASTAT 2.9.3.2 and GENEPOP 3.4 (Raymond & Rousset, 1995), respectively. We used Bonferroni correction to adjust the significance values for multiple comparisons.

### 2.4 Genetic structure analysis

A Bayesian clustering approach, implemented with STRUCTURE v2.3.4 (Pritchard, Stephens, & Donnelly, 2000), was used to detect genetic structure within the Qinling giant panda population. Clustering solutions of *K *=* *1–10 were tested using an admixture model with correlated allele frequencies. For each simulation, 10^6^ Markov Chain Monte Carlo (MCMC) iterations were used after 10^5^ burn‐in simulations. Each value of *K* was repeated using 20 independent runs. The log likelihood of the posterior probability (LnPr(X|K)) and its rate of change (ΔK) were both evaluated to infer population clustering.

### 2.5 Spatial autocorrelation analysis

The Mantel correlogram was used to compute spatial autocorrelation with genetic structure at a fine spatial scale, and Mantel test was also performed to identify the IDB pattern in the population. Both analyses were implemented in GenAlEx 6.5 (Peakall & Smouse, 2012), with genetic distance between each pair of individuals was estimated using the proportion of shared alleles calculated in MSA V4.05 (Dieringer & Schlotterer, 2003). Based on three long‐term radiotelemetry and GPS‐telemetry studies (Schaller, Hu, & Pan, 1985; Pan et al. 2001; Zhang et al., 2014), the radius of mean home range has been estimated as 1.9 km. We used this value to define distance classes in the analysis, and we analysed up to fifteen distance classes among samples.

### 2.6 Isolation‐by‐resistance (IBR)

In order to decipher the role of each landscape factor in shaping population genetic structure in giant pandas, we analysed the relationship between eight landscape variables—elevation, slope aspect, slope, topographic complexity (TC), rivers, vegetation, roads and human disturbances, with genetic differentiation patterns based on an IBR model (McRae, 2006).

First, a simple resistance surface representing the distance effect alone (referred to as the model IBD in the following text) was constructed by setting a value of “1” to all the raster cells using CIRCUITSCAPE V3.5 (McRae, Shah, & Mohapatra, 2013), and the partial Mantel tests were evaluated by partialling out this resulting resistance matrix. Genetic distance was measured as the proportion of shared alleles calculated in MSA V4.05 (Dieringer & Schlotterer, 2003). A Euclidian distance matrix was estimated in GenAlEx V6.5 (Peakall & Smouse, 2012). In addition, resistance surfaces for each landscape factor were obtained depending on specific ecological hypotheses, under the pairwise mode connecting eight neighbours based on the average resistance implemented in CIRCUITSCAPE V3.5. For the full descriptions of model construction see Supporting Information.

Although Mantel and partial Mantel tests have been widely used in correlating the landscape pattern with gene flow, these tests have been shown to possess a high risk of spurious correlations. Several refinements, including causal modelling, have been developed to reduce this problem. The approach involving direct competition of all hypotheses proposed by Cushman, Wasserman, Landguth, and Shirk (2013) could lessen the type I error rate which has been found to be high in the original causal modelling analysis. By combining the causal modelling approaches proposed by Cushman et al. (2013) and Wasserman, Cushman, Schwartz, and Wallin (2010), partial Mantel tests could be reliable in estimating the correlation between landscape variables and gene flow.

Partial Mantel tests were carried out between each landscape resistance surface matrix with the genetic distance matrix. A landscape factor was recognized affecting the genetic differentiation pattern above that of the null model (the model IBD) when the following criteria were met: (a) the partial Mantel test between genetic distance and landscape resistance model must be significant when the effect of IBD was excluded (GD~LR|IBD); (b) the partial Mantel test between genetic distance and the model IBD was not significant when the landscape resistance effect was excluded (GD~IBD|LR). A model could be supported independently of other candidate models if (c) the partial Mantel test between genetic distance and the true landscape model was significant when the effect of the reduced model was excluded (GD~LM|RM) and (d) the partial Mantel test between genetic distance and the reduced model was not significant when the effect of the landscape model was excluded (GD~RM|LM).

Rather than simply competing the alternative landscape models against model IBD, a set of candidate models with similar parameters as the top model were assessed against each other by estimating their relative support (RS). Relative support can be quantified as: RS_1|2_ = GD~LR_1_|LR_2_ – GD~LR_2_|LR_1,_ where the LR_1_ represented the resistance matrix of landscape variable 1 and LR_2_ represents the resistance matrix of landscape variable 2. The GD~LR_1_|LR_2_ is the partial Mantel correlation between genetic distance and landscape variable 1 when partialling out the effect of landscape variable 2. The best candidate model should therefore possess positive RS value for all comparisons and has to pass the causal modelling criteria against the model IBD.

Considering the complex landscape giant pandas live in, a multivariate approach was also used to explain gene flow within this population. The multivariate resistance surface matrix was equal to the sum of univariate resistance matrices of each landscape variable. We started with a simple model with only two landscape variables which had the highest partial Mantel *r* value when partialling out the effect of the model IBD. We kept the parameter for the first variable stable while altering the parameter values for the second variable to create a series of bivariate models. The best parameter for the second landscape variable was identified by comparing the partial Mantel *r* value removing the effect of the model IBD of bivariate models with each other. Then, we kept the parameter values for the second landscape variable constant but altered the parameter values for the first landscape variable to obtain the best parameter for the first landscape variable in a similar way. We re‐optimized the remaining variables by adding them one at a time to the initial bivariate model until the parameter set for the best‐supported model did not change. Multivariate models were also evaluated using their RS values, by altering the model parameters for one variable while holding the others constant until variable parameters did not change. In order to be accepted, the multivariate model had to pass the two‐step criteria of the causal modelling to show it was better than model IBD. In addition, a landscape variable was excluded from the best‐supported multivariate model, if it did not pass the causal modelling criteria with a reduced model, if the GD~(A+B+C)|(A+B) model was significant, and the GD~(A+B)|(A+B+C) was not significant.

### 2.7 Mixed‐effect models

We also implemented a linear mixed‐effect model analysis to investigate the influence of landscape variables on the gene flow using the “lme4” package in R (Bates, Maechler, Bolker, & Walker, 2015). To correct for the dependency among data points, a maximum‐likelihood population‐effect (MLPE) method was followed (Clarke, Rothery, & Raybould, 2002; Van Strien, Keller, & Holderegger, 2012). In these models, differences between sampling units were introduced as random‐effect terms, while the explanatory variables were introduced as fixed‐effect terms, which we were most concerned. The parameter set for the MLPE model was fitted with the residual maximum‐likelihood (REML). After standardizing all the explanatory variables, the REML estimates of the intercept were the same as estimates obtained from simple linear regressions. Explanatory variables with variance inflation factors (VIF) above 5 were suspected of collinearity and excluded from models. We built a full model with all the variables without significant collinearity to identify the combined effects of multiple variables on gene flow. This full model was refined using “MuMIN” package in R (Bartoń, 2018). We compared the corrected Akaike Information Criterion values (AICc) and AICc weights (*wi*) among models averaged from the global model to identify the best model. $R_{\beta }^{2}$ *,* which compares a model with fixed effects to a null model (we used the model IBD as our null model) with only random effect and an intercept, was also calculated, using Kenward‐Roger *F* and degrees of freedom, with the “KRmodcomp” function from the R package “pbkrtest “(Halekoh & Højsgaard, 2014). We used the $R_{\beta }^{2}$ to indicate the proportion of variance explained by the models.

### 2.8 Current density map and potential corridors

We also formulated a current density map using CIRCUITSCAPE V3.5 based on the best‐supported hypothesis. The density of current flow in an area reflects its connectivity and movement possibility for the animal. Thus, ecological corridors that will promote gene flow effectively can be identified based on this information. Several quantitative criteria were also followed when we identified the optimal position of a corridor in this study: (a) Degree of overlap with the area featuring the lowest resistance and highest current density. Overlapping with low resistance area, corridors could provide giant pandas with even and continuous pathways facilitating their dispersal and movement. (b) Reconnecting populations divided by major roads. There are several major roads running through the Qinling Mountains, which markedly divide and isolate habitat patches and local populations. To counter this barrier effect and promote gene flow, corridors should cross the major roads to connect neighbouring habitat patches. (c) Avoid farmlands by more than 1 km and residences by 2 km. Human activities impede utilization of corridors. According to a GIS study on giant pandas at different spatial scales, anthropogenic activities still disrupt the animals’ movements at a relative large range. In particular, farmland impedes giant pandas’ movements at a 1 km range, while the residences have larger disturbance range at 2 km (State Forestry Administration 2015). These criteria could act as the model to design corridor for the giant panda in other regions.

## 3 RESULTS

### 3.1 Genetic variation and genetic structure

**Figure 1:**
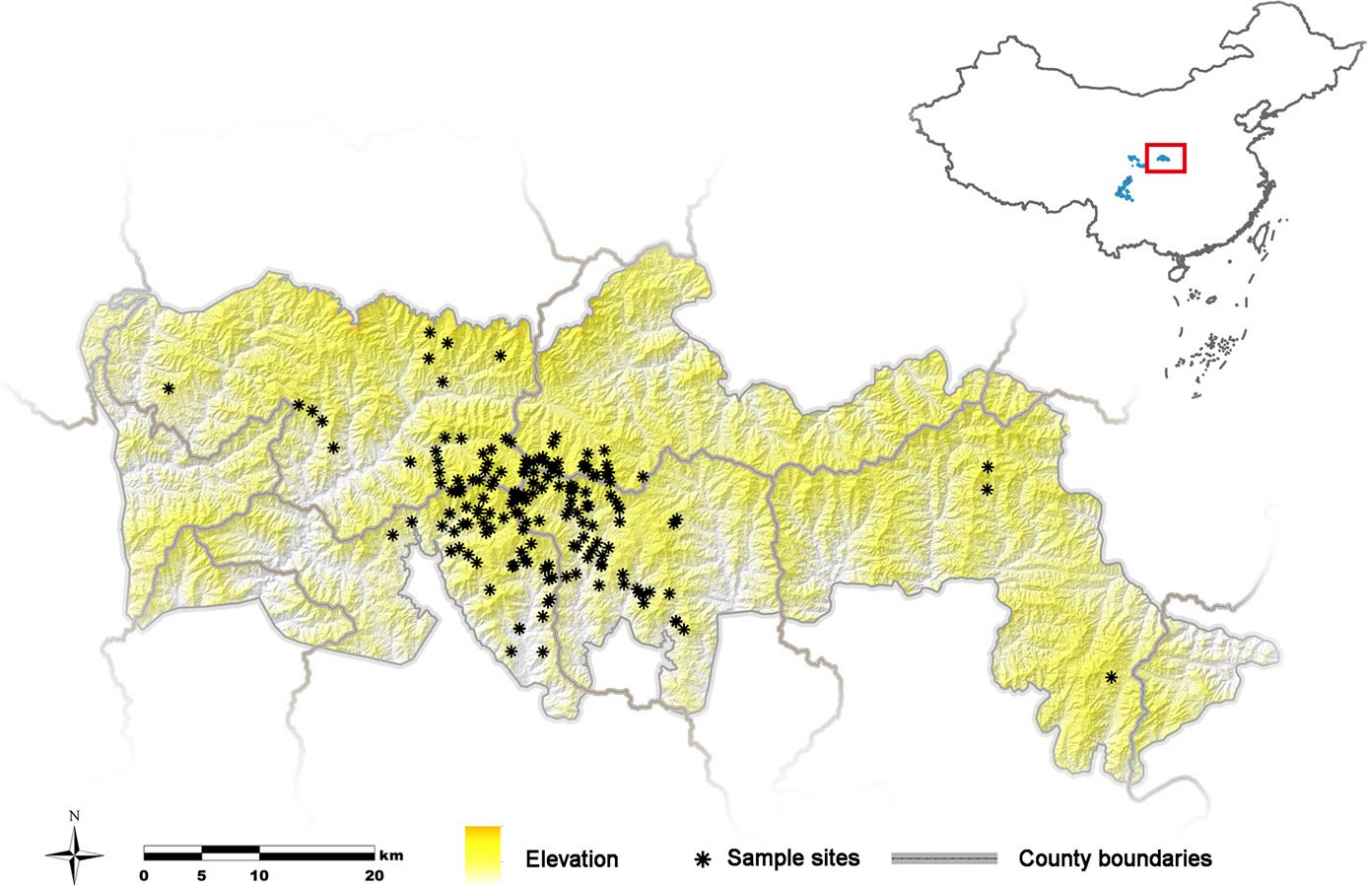
Map of study area with locations of 178 giant panda individuals

A total of 285 multilocus genotypes were obtained from 534 faecal samples, with 178 unique individuals being identified (Figure 1). The set of 12 microsatellite loci used in this study were estimated to produce random identical genotypes with a probability of 1.2 × 10^−8^, with a probability of 3.6 × 10^−4^ for full‐sibs. We used samples with at least nine loci genotyped successfully to guarantee the reliability of subsequent analyses. MICRO‐CHECKER analysis indicated there was no evidence of null alleles, large allele dropout or stuttering in this data set. No consistent departures from Hardy–Weinberg equilibrium or linkage disequilibrium were detected after Bonferroni correction. Using 12 microsatellites, a mean number of 4.58 alleles per locus was estimated, and observed (H_O_) and expected heterozygosity (H_E_) were 0.488 and 0.447, respectively, similar to previous studies (Lu et al., 2001; Zhang et al., 2007), but lower than that of other giant panda populations (Supporting information Table S2).

STRUCTURE analysis showed that the most likely number of cluster based on LnPr(*X*/*K*) was *K *=* *1. When *K *>* *1, the proportion of the individual assigned to each cluster was approximately equal, indicating there was no genetic structure in Qinling giant panda population (Supporting information Figure S1). Overall, the Qinling giant panda population showed no evidence of fine‐scale population substructure.

### 3.2 Spatial autocorrelation

**Figure 2:**
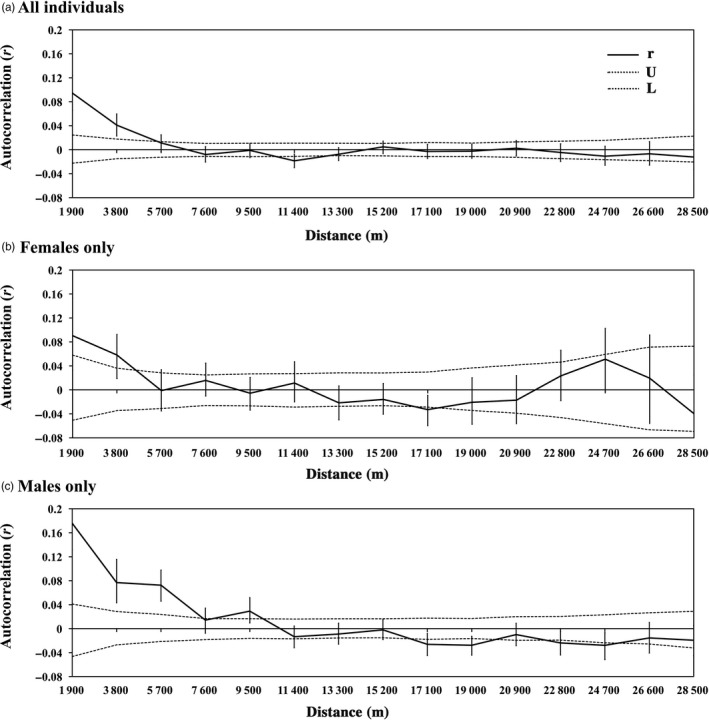
Spatial autocorrelograms of all giant pandas, all females and all males. Spatial autocorrelograms of genetic correlation coefficient (*r*) as a function of geographical distance, with the permuted 95% confidence intervals (dashed lines) indicating random spatial genetic structure and the bootstrapped 95% confidence error bars around *r*. a) All giant panda individuals (*n *=* *179); b) females only (*n *=* *102); c) males only (*n *=* *59)

The spatial autocorrelation analysis including all individuals suggested local genetic structure within the study area. Based on the Mantel correlogram, a significant positive correlation between the pairwise genetic distance and Euclidean distance occurred in the first two distance classes (1–1900 m and 1901–3800 m, Figure 2a). This correlation was not significant in all other distance classes, with one distance class showing a negative correlation (Figure 2a). However, a simple Mantel test of IBD implemented in GenAlEx 6.5 found no significant correlation (*p *=* *0.444) between pairwise genetic distance and Euclidean distance in our sample set, indicating that IBD did not play a key role in the genetic differentiation pattern of the Qinling giant panda population. Compared to the females showing significant correlations in the first two distance classes, significant positive correlations in males were still found until 9.5 km (except for the fourth distance class; Figure 2b,c). In addition, the *r* value for males in first distance class was nearly twice the result for females (female: 0.09; male: 0.176), although the bootstrap error bars showed the *r* value for males was not significantly greater than that for females.

### 3.3 Isolation‐by‐resistance

A total of 534 models for eight landscape variables with different parameters were processed to detect their relationship with genetic distance. Ninety slope models were excluded, because the Mantel correlations between them and genetic distance were not significant (*p *>* *0.05). By ranking the partial Mantel *r* values of different models, slope aspect was found to be most correlated to genetic distance after partialling out the effect of the model IBD (Table 1). Topographic complexity had the second highest partial Mantel *r* value followed by elevation, vegetation and the rest landscape factors (Table 1). When these models were optimized based on relative support (RS), the parameter values did not change much, and only aspect and TC met the casual modelling criteria, which would be finally included into the multivariate model construction (Table 2).

**Table 1:** Best univariate models of landscape resistances based on partial Mantel correlation after partialling out the effect of the IBD model

Landscape variable	Parameter values	Partial Mantel *r*	*p*‐value
Aspect	*90°; x *=* *10*; R*_max_ = 500	**0.103**	**0.0229**
TC	*x *=* *10*; R*_max_ = 500	**0.09204**	**0.0374**
DEM	2800 m; *R*_max_ = 1000; SD = 200	0.05781	0.1133
Vegetation	Assigned based on a permutation	0.05622	0.1324

*Notes*

Models are ranked with the partial Mantel *r*‐value. Optimized parameter values, partial Mantel *r* and significance of support are displayed. Supported models are indicated in bold. DEM: digital elevation model; TC: topographical complexity.

**Table 2:** Models are ranked with the best‐supported model at the top

Landscape variable	Parameter values	RS_IBD_	(A)*r*	(A)*p*	(B)*r*	(B)*p*	Supported
Aspect	90°; *x *=* *10; *R*_max_ = 100	0.12793	0.09897	0.026	−0.02896	0.739	**YES**
TC	*x *=* *4; *R*_max_ = 100	0.11661	0.0911	0.0358	−0.02551	0.6962	**YES**
DEM	2800 m; *R*_max_ = 1000; SD=200	0.10764	0.05781	0.1133	−0.04983	0.8769	**NO**
Vegetation	Assigned based on a permutation	0.09329	0.05622	0.1324	−0.03707	0.7949	**NO**

*Notes*

Optimized parameter values, RS (relative support) value as compared to IBD, partial Mantel *r* and significance of support are shown. Optimized values include equation parameters for *x* (contrast) and *R*_max_ (magnitude of the relationship). (A) GD~LV|IBD—partial Mantel test between genetic distance and landscape variable, partialling out the effect of IBD; (B) GD~IBD|LV—partial Mantel test between genetic distance and IBD distance, removing the effect of the landscape variable. The first column of each test indicates the Mantel *r*‐value and the second column the related *p*‐value. Supported models are indicated in bold. DEM: digital elevation model; TC: topographical complexity.

By changing the parameter values of either factor and keeping that of the other one constant, we finally found that the best‐supported multivariate model included aspect and TC, removing the effect of the model IBD (Table 3). The parameter values of aspect and TC in this optimized multivariate model coincided with the parameter values of their best univariate models. We also tested the causal modelling criteria with two reduced models (equalled to the best univariate model of aspect and TC, respectively) to this best multivariate model, and both reduced models passed the criteria. When comparing the partial Mantel correlations of reduced models with the best multivariate model, we found the reduced models were both improved by including the other factor (the *r* value increased from 0.082 to 0.163 after including aspect, and from 0.098 to 0.163 after including TC); namely, more variance in pairwise genetic distance could be explained by including aspect and TC at the same time. Based on the above results, we suggested that aspect and TC more predominantly influenced gene flow within the Qinling giant panda population than other six factors.

**Table 3:** The Optimized parameter values, RS value as compared to IBD, partial Mantel *r* and significance of support of the best multivariate model are shown

Model	Parameter values	RS_IBD_	(A)*r*	(A)*p*	(B)*r*	(B)*p*	(C)*r*	(C)*p*	(D)*r*	(D)*p*
A+TC	A: 90°; *x *=* *10; *R*_max_ = 500	0.16288	0.1324	0.0045	‐0.03048	0.7482	A:0.09791	0.0286	A:0.03225	0.2599
	TC: *x* = 10; *R*_max_ = 500						TC:0.08123	0.0515	TC:‐0.01118	0.5949

*Notes*

Optimized values include equation parameters for *x* (contrast) and *R*_max_ (magnitude of the relationship). (A) GD~LV|IBD—partial Mantel test between genetic distance and the landscape variable, partialling out the effect of IBD; (B) GD~IBD|LV—partial Mantel test between genetic distance and IBD distance, removing the effect of the landscape variable, (C) GD~LM|—partial Mantel test between genetic distance and the landscape model after removing the effect of the reduced model; (D) G~|LM—partial Mantel test between genetic distance and the reduced model, partialling out the effect of the landscape model. The first column of each test indicates the Mantel *r*‐value and the second column the related *p*‐value. Model abbreviations: A: aspect and TC: topographic complexity.

### 3.4 Mixed‐effect models

By evaluating the performances of a series of MLPE models, the geographic distance resistance surface did not explain the variance in genetic distance ($R_{\beta }^{2}$ < 0.01%), suggesting that the geographic distance is not the main force driving gene flow in the Qinling giant panda population. Two variables, the model IBD and slope, were excluded from the full model due to collinearity. The best model included two variables, aspect and topographic complexity (TC; AICc = −23446.40, *wi *= 0.73, Table 4), and thus, these two variables corresponded to the best‐supported hypotheses under causal modelling criteria. While other models were different from the best model by more than three AICc units, we identified that models B, C and D were partially supported for the differences between them and the best model were within seven AICc units (Table 4). Based on the $R_{\beta }^{2}$ values of the top models, the performance of models in explaining the variance in genetic distance could be slightly improved when adding retained landscape variables into the reduced model. Model D including aspect, TC, roads, vegetation and elevation explained the highest proportion of the variance in genetic distance (Table 4). These results suggested that the topographic factors, such as slope aspect and topographic complexity, played an important role in shaping gene flow patterns in the population, while anthropogenic disturbance such as roads had a smaller effect on gene flow.

**Table 4:** Mixed‐effect models show the correlation between pairwise genetic distance and resistance distance of different landscape variables

Model	Type of model	Variables	VIF	$R_{\beta }^{2}$	AICc	∆AICc	Weight (*w*_*i*_)
A	Reduced	Aspect	1.02	0.008	−23446.40	0.00	0.73
		TC	1.03				
B	Reduced	Aspect	1.02	0.008	−23443.30	3.04	0.16
		TC	1.03				
		Road	2.39				
C	Reduced	Aspect	1.02	0.009	−23440.30	6.13	0.03
		TC	1.03				
		Road	2.39				
		Vegetation	1.22				
D	Reduced	Aspect	1.02	0.010	−23440.10	6.30	0.03
		TC	1.03				
		Road	2.39				
		Vegetation	1.22				
		Elevation	1.93				
E	Reduced	Aspect	1.02	0.009	−23438.40	8.02	0.01
		TC	1.03				
		Road	2.39				
		Elevation	1.93				

*Notes*

To minimize colinearity among predictors, all variables with VIF values > 5 were removed. VIF: Variance Inflation Factor. The best‐fitting model was selected based on the corrected Akaike information criterion (AICc, ∆AICc, *wi*). We used *R* ^*2*^ statistics ($R_{\beta }^{2}$) to describe the amount of variation explained by the model. Models with the highest AICc support are in bold (∆AICc ≤ 2). Marginally supported models are also indicated (∆AICc ≤ 7). TC: topographical complexity.

### 3.5 Current density map and potential corridors

**Figure 3:**
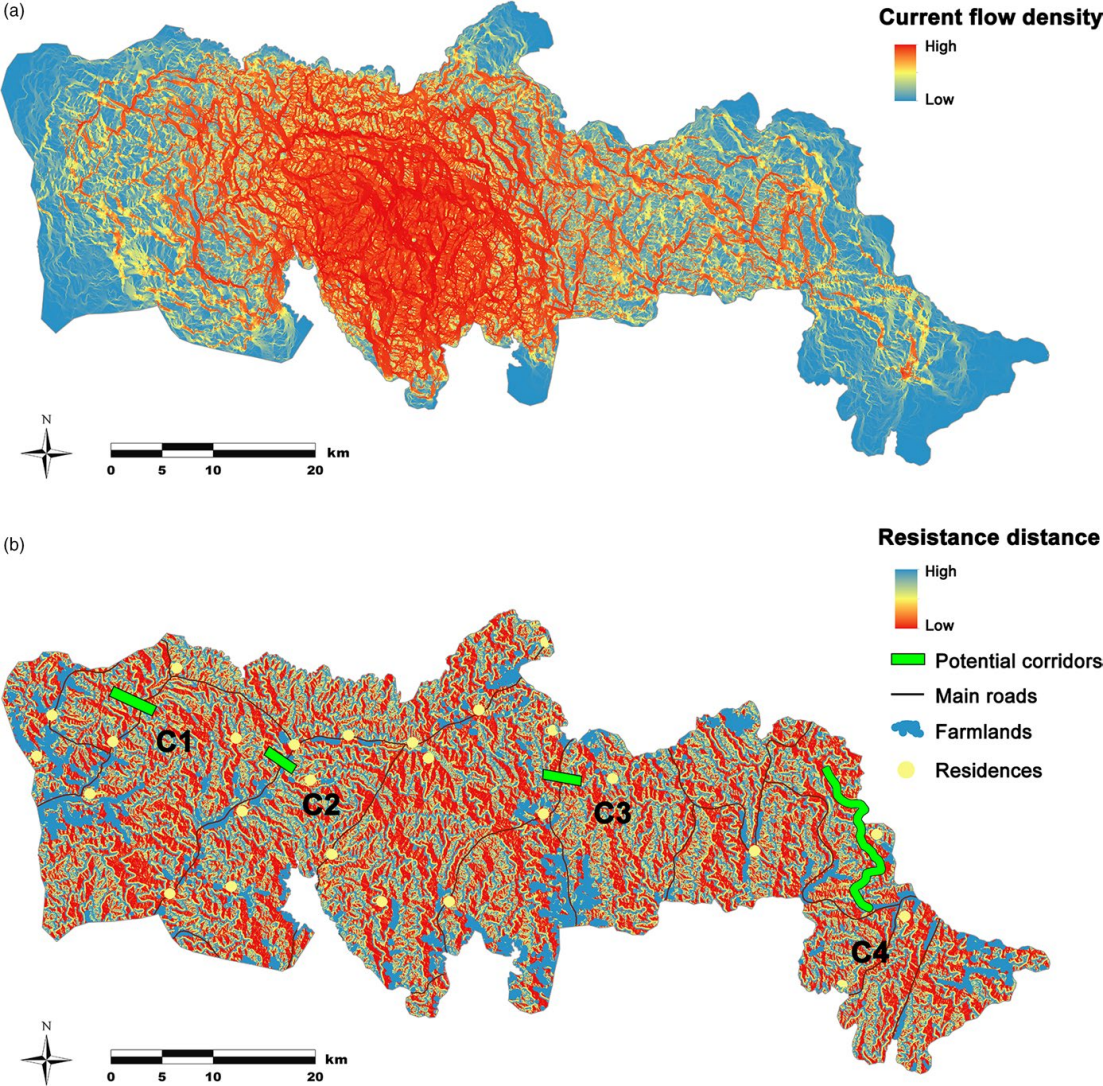
Maps of the current density and potential corridors in the study area. a) The current map was generated by CIRCUITSCAPE V3.5, and displayed by histogram equalization. The areas with the highest current density representing the highest connectivity are shown in red while the lowest are shown in blue colour. b) The resistance surface map based on the best hypothesis, Aspect + TC, about the gene flow, with the information of roads and human disturbances also shown. The proposed best position for corridor between adjacent habitat components are highlighted with green, with Corridor C1, C2, C3, C4 connected TBH + NWH, NWH + XT, XT + TJ, TJ + PHL, respectively

Areas of high current density are represented in red in Figure 3a, showing a netlike distribution with spatial heterogeneity across the study region. There is a distinct concentration of strong current flow in the central part of the study area, possibly due to more intense sampling efforts and closer distances between samples.

Habitat connectivity is essential for gene flow and the sustainability of animal populations. Through the current density map, we could evaluate habitat connectivity and assess potential areas for corridor construction which would increase genetic exchange between populations in divided habitat patches. Previous mountain‐scale surveys on the giant panda have suggested that there are five habitat components including one or more patches in this study area, named Taibaihe (TBH), Niuweihe (NWH), Xinglongling–Taibaishan (XT), Tianhuashan–Jinjiliang (TJ) and Pingheliang (PHL) from west to east. These habitat components are separated by major roads and human activities. Solely based on the current density map, we could identify multiple narrow high current density pathways as candidates for potential corridors to connect habitat components. However, frequent anthropogenic disturbance could hamper corridor utilization by animals and diminish its efficiency in promoting gene flow. Thus, according to three quantitative criteria established in this study, we finally identified an optimal corridor for each pair of adjacent habitat components (Figure 3b), which can efficiently facilitate gene flow in the giant panda population from the area.

## 4 DISCUSSION

### 4.1 Lowest genetic diversity recorded of all giant panda populations to date

Here, we report the first comprehensive study to explore the population and landscape genetic structure of the Qinling giant pandas, with a large noninvasive genetic sample set. We found the lowest genetic diversity in the Qinling population among all panda populations, consistent with our hypotheses and the conclusions drawn by previous studies using similar molecular marker (Lu et al., 2001; Zhang et al., 2007).

The relatively low genetic diversity of the Qinling population may be associated with its demographic history. The Qinling giant panda population has been inferred to have experienced a severe bottleneck in the Late Pleistocene (Pan, Gao, & Lu, 1988; Pan et al., 2001; Zhao et al., 2013), with an 80% decline in effective population size being suggested by a recent genomic study (Zhao et al., 2013). Intense anthropogenic habitat alteration in the Holocene has triggered further population decline and likely further loss of genetic diversity in the Qinling population. Therefore, we suggested that the Qinling population's low genetic diversity has been driven by continuous population decline and long‐term isolation, also increasing its divergence from the Sichuan population.

### 4.2 Spatial genetic pattern of Qinling giant pandas: IBB, IBD or IBR?

Previous ecological studies have suggested that the Qinling population was a metapopulation composed by four to five subpopulations isolated by farmlands, deforested areas, plantation forests and national roads (Loucks et al., 2003; State Forestry Administration 2006). However, we did not find evidence for significant IBB effect in this population based on a Bayesian genetic clustering approach. Commercial logging used to be common in the Qinling Mountains which also boosted road construction and housing construction during the 1970–1990. During this period, suitable habitat for giant pandas must have declined, with several boundaries hampering dispersal being formed. However, as local and national authorities paid more attention to environmental and wildlife protection, a logging ban in natural forests since 2000 and the construction of tunnels under several sections of traditional road have mitigated against additional disturbance to the Qinling habitat (Li et al., 2013; Swaisgood, Wang, & Wei, 2017) and obstructions to gene flow. The lack of evidence for genetic structure within the region could be due to the relatively short timescale involved in habitat patch separation, with giant pandas still being able to use some of the less disturbed regions as corridors to move between patches. It is worth noting that the limited sampling of the two easternmost hypothetical subpopulations affects these results, although the 178 individuals included comprise more than 50% of the estimated population size in the Qinling Mountains (345 individuals; Fourth National Survey of Giant Pandas).

We only detected significant genetic correlations between individuals in close distance classes, indicating that IBD does not explain spatial genetic structure of giant pandas in Qinling Mountain, a result confirmed by the Mantel test result (*p *=* *0.44). One of the primary reasons for this observation might be natal dispersal pattern in giant pandas. In our analysis, male giant pandas represented closer genetic relationships than females in proximal distance. This finding is in accordance with a recent study in giant pandas (Hu et al., 2017). Some long‐distance dispersal events have been recorded in Qinling (Pan et al., 2001; Zhang et al., 2007), and this kind of behaviour is expected to disrupt the IBD pattern.

Wild giant panda habitat includes complex environment components. Landscape factors including extreme topographic and vegetation transitions are considered to affect giant panda movements and habitat selection (Hu, Schaller, Pan, & Zhu, 1985; Reid & Hu, 1991; Wei, Feng, Wang, & Hu, 2000; ;Zhang et al., 2011; Qi et al., 2012), but the impact of these factors on the spatial pattern of genetic variation has not been separately examined. The only landscape genetic study on giant pandas to date was on small and isolated populations in the Daxiangling and Xiaoxiangling Mountains (Zhu, Zhang, Gu, & Wei, 2011; Zhu et al., 2010). In the study, a large river and a road were found to be barrier impeding gene flow of giant pandas in the region. The authors also suggested that some landscape features partially influenced gene flow based on least‐cost path analysis. However, conclusions on how specific landscape variables impacted gene flow were not possible.

We investigated eight key landscape features using a relatively large genetic data set, and ultimately, slope aspect proved to be strongly associated with genetic variation in the Qinling population, followed by topographic complexity. Qinling giant pandas appear to have a preference for the east‐facing slopes. This preference has also been found in brown bears from the Carpathians Mountains, considered to be related to food availability in the breeding season (Cotovelea, 2014), and other small mammals (Castillo, Epps, Davis, & Cushman, 2014; Russo, Sole, Barbato, von Bramann, & Bruford, 2016). Relatively flat (topologically simple) ground seems ideal for giant panda dispersal behaviour, while complex topography is avoided. This is compatible with giant pandas’ low energy budget, given its diet and its need to conserve energy (Nie et al., 2015).

The south side of the Qinling Mountains provides suitable habitats to sustain a high‐density giant panda population. This habitat is located in central China and is in the East Asian Monsoon Region. The continuous ridge of the Qinling Mountains obstructs cold air from the north and traps warm and wet monsoon air from the Pacific Ocean in summer, thus providing suitable climatic conditions for giant pandas. At a finer scale, east‐facing slopes not only have sufficient solar radiation in the morning, but also avoid overtranspiration in the afternoon, which helps to keep a suitable microclimate for the development of bamboo. Previous field surveys have also shown that bamboo forest on east/south‐facing slopes grow faster and accumulate biomass more quickly (Pan et al., 2001).

Topographic complexity is related to a series of habitat conditions with variable solar radiation and soil moisture, possibly influencing plant growth and hindering animal movements, but has seldom been considered in previous ecological research on giant pandas. Here, we could infer that giant pandas use less complex land surfaces, different from conclusions for some other bear species which have been suggested to prefer areas with complex topographies (Apps, McLellan, Woods, & Proctor, 2004; Ziółkowska et al., 2016). Complex terrain is commonly associated with better availability of heterogeneous food resources, sheltering opportunities and implies less human disturbance (Ziółkowska et al., 2016). However, as an exclusive bamboo‐eater, the giant pandas’ requirements seem much simpler. Heterogeneous vegetation composition does not imply ampler food resources and may obstruct movement. Furthermore, microtopographic structure could result in more complex shading effects, while less complex surfaces are expected to provide more abundant and even sunlight, more compatible with bamboo growth. To survive on their poor‐nutritional bamboo diets, giant pandas possess a set of strategies to balance energy budgets. Avoiding the extra energy expenditure needed for moving over difficult terrain is compatible with the giant pandas’ energy‐economy strategy, while their nutritional requirements are met at the same time. The habitat and movement preference of Qinling giant pandas corresponds to the species’ biological and ecological requirements. The difference in the preference for complex landscapes between the giant panda and some other bear species reflects the variation of environmental needs due to unique evolutionary processes. These findings highlight the species‐specific empirical studies on this issue and could improve the efficiency of management and conservation planning, especially for endangered species.

Concluding the results of IBB, IBD and IBR tests, gene flow was significantly influenced by only two landscape factors over the geographic scale defined in this study. By evaluating different landscape genetic hypotheses, a relatively comprehensive understanding of the key factors shaping genetic structure and gene flow can be achieved. Multihypothesis approaches enable researchers to interpret the relationship between spatial heterogeneity and population genetic variation at more precise spatial scales. This is crucial for the conservation of endangered animal populations, considering that strategies based on incorrect inferences could result in the waste of limited conservation resources and, most importantly, may miss the opportunity to retrieve critically endangered populations. In consequence, more and more landscape genetic studies employ this approach. However, the number of empirical studies that have fully adopted it remains few (but see Cushman & Landguth, 2010; Cushman et al., 2014; Yang, Cushman, Song, Yang, & Zhang, 2015; Ruiz‐Gonzalez et al., 2015). Taking into consideration the clear advantage of this approach, we promote wider awareness and application of multihypothesis approaches in landscape genetic research, especially on endangered species.

### 4.3 Conservation implications

We did not detect any genetic substructure in the Qinling population so far, which is important given that this giant panda population already has relatively low genetic diversity due to its past demographic history. However, roads and logging continue to fragment the Qinling Mountains, and are ultimately likely to impact on gene flow to some extent. If no action is taken, the legacy of previous actions and future infrastructure development could result in the demographic isolation among local patches, and impact on the long‐term survival of this unique giant panda population.

In this context, we identified several corridors to connect the key habitat components in order to promote gene flow within this unique giant panda population. Among five habitat components, XT sustains the largest local population, playing the role of demographic “source” in the region (Pan et al., 2001). However, the Yangtai road and national road G108 have hindered the exchange of individuals in XT with other habitat components, leading to the rise of XT population and that may exceed theoretical carrying capacity (Gong, Yang, Yang, & Song, 2010). We identified several east–west banded zones, that are topographically less complex, as candidates for corridors. However, human disturbance including farming and settlements could hamper giant panda utilization for corridors, limiting the options for corridor selection. At last, we identified C2 and C3 as the optimal corridors connecting XT to its neighbouring habitat components, NWH and TJ, respectively. These corridor positions are similar to some are in previous studies based on ecological habitat evaluation data (Gong et al., 2010; Wang et al., 2014), but more localized. While most corridor studies focused on habitat patches in the middle of the Qinling Mountains where the majority of Qinling giant pandas live, the patches close to the edge have attracted less attention. However, during the latest mountain‐scale survey, new records of giant panda activity were discovered in TBH located in the western edge of the region (State Forestry Administration 2015). As logging and housing become more restricted in the Qinling Mountains, giant pandas could be able to re‐utilize the habitat patches with previously high level of human disturbance. Corridor C1, connecting the TBH and NWH, could assist more individuals in dispersing to newly available habitat while expanding the total area of suitable habitat. PHL is located in the easternmost part of the Qinling habitat, sustaining a small population consisting of only seven individuals (State Forestry Administration 2015). Its isolated nature and intense anthropogenic influence put the PHL population at high risk of extinction. Increasing its connectivity with other populations is vital for its long‐term survival. However, while a nature reserve has already been established between PHL and the adjoining habitat patches, it currently does not include the zones with the highest potential to facilitate giant panda dispersal, as identified in our study (corridor C4). Therefore, in future conservation planning, the nature reserve should be expanded or modified to cover these key linkage zones to ensure an effective connection between PHL and its neighbouring habitat patches. In addition, the reintroduction of captive individuals to this isolated population should be considered. Furthermore, it is important that, to retain the genetic uniqueness of the Qinling population, managers should carefully consider the genetic background of released individuals. Only with the establishment of key linkage zones, conservation measures and a reasonable reintroduction plan, will this small isolated population have a chance of rejuvenation and long‐term survival.

## CONFLICT OF INTEREST

None declared.

## DATA ACCESSIBILITY

The microsatellite data available from the Dryad Digital Repository: https://doi.org/10.5061/dryad.5sh56g0
