# Supplementary material for: Walking in a heterogeneous landscape: Dispersal, gene flow and conservation implications for the giant panda in the Qinling Mountains
Source: Evol Appl. 2018 Aug 31;11(10):1859–72. doi: 10.1111/eva.12686 (PMC6231463; doi:10.1111/eva.12686)
Supplement: Supplementary file 1 [file EVA-11-1859-s001.doc]

**Supplementary Information**

**Walking in a heterogeneous landscape: dispersal, gene-flow and conservation implications for the giant panda in the Qinling Mountains**


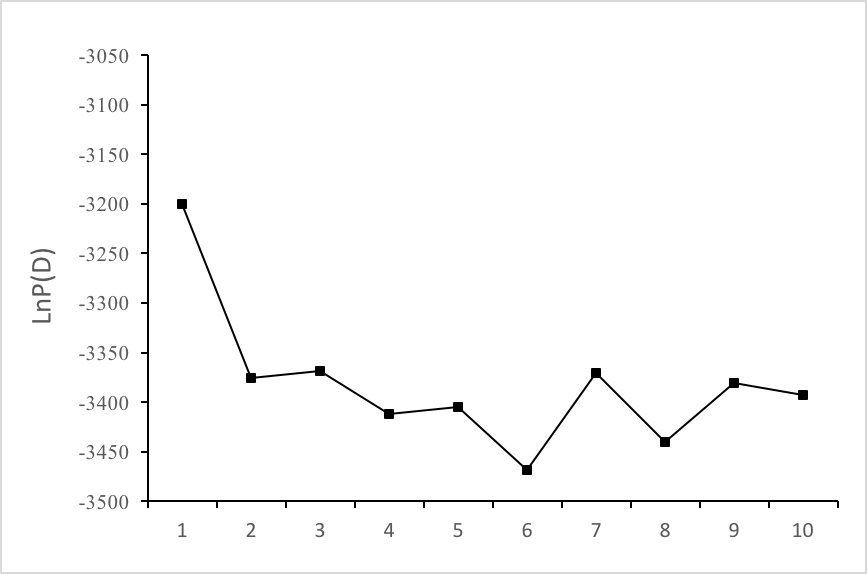


**Fig. S1** Estimated number of clusters inferred using Bayesian clustering analysis performed with STRUCTURE for the whole dataset. Black squares indicate the mean probability log-likelihood (LnP(D)) from 20 repeats for each value of *K*. The peak of LnP(D) appeared at *K* = 1.

**Table S1** Attributes of the 12 giant panda-specific microsatellite loci used in this study.

| **Locus** | **Primers** | **Size of repeat motif** | **Product size range** | **Annealing temperature (℃)** | **Fluorescent dye** | **Reference** |
| --- | --- | --- | --- | --- | --- | --- |
| Ame-μ10 | 5’-ACC GTG CTC TTA ATC CCC TT-3’ | 2bp | 134-150 | 48 | HEX | Lu et al. (2001) |
|  | 5’-CCC ATG CTT ATG AGA AAC AGG-3’ |  |  |  |  |  |
| Ame-μ11 | 5’-CTC TTT GCA TCT CAA ATT CA-3’ | 2bp | 108-116 | 48 | TAMRA | Lu et al. (2001) |
|  | 5’-TGA GAT AGG AAA GAG TGG GT-3’ |  |  |  |  | Wu et al. (2009) |
| Ame-μ13 | 5’-GGA AGC ATT AAG GAA AAC ATG C-3’ | 2bp | 138-160 | 50 | HEX | Lu et al. (2001) |
|  | 5’-AAT GAT GAC CAT TTC AAA CGC-3’ |  |  |  |  |  |
| Ame-μ15 | 5’-AAG CAG TTG TTT TTG CTT AGT G -3’ | 2bp | 120-124 | 48 | TAMRA | Lu et al. (2001) |
|  | 5’-TGT CAA AGT ATT TGC CTC ACA-3’ |  |  |  |  |  |
| Ame-μ22 | 5’-AGG AAA CAT GTT GCC TTT TCA-3’ | 2bp | 127-131 | 50 | FAM | Lu et al. (2001) |
|  | 5’-AGA GGG CAA ATA GGA GGG AA-3’ |  |  |  |  |  |
| Ame-μ24 | 5’-TAT TTG TCA TGG GGT TTA CT-3’ | 2bp | 113-119 | 50 | FAM | Lu et al. (2001) |
|  | 5’-CCT ACC TCA CAT TCT ACT CC-3’ |  |  |  |  | Wu et al. (2009) |
| Ame-μ26 | 5’-TTT TCA GGC CTC CGA AAA C-3’ | 2bp | 108-114 | 48 | FAM | Lu et al. (2001) |
|  | 5’-ATT CCC AAT AAA GCA AAT CAG A-3’ |  |  |  |  |  |
| Ame-μ27 | 5’-TTG AAG AAG AAG GAA CAT TCC C-3’ | 2bp | 128-144 | 48 | FAM | Lu et al. (2001) |
|  | 5’-TTT RGC AAC TAT GTC CCT CAG G-3’ |  |  |  |  |  |
| AY79 | 5’-GAG CAC TCT TGG GTT TTG TTA-3’ | 2bp | 142-158 | 48 | TAMRA | Shen et al. (2005) |
|  | 5’-TGG TGG GCA GGA ATG AT-3’ |  |  |  |  | Wu et al. (2009) |
| AY95 | 5’-GCA TGA AAG CCA GAA AAC AG-3’ | 3bp | 142-148 | 48 | FAM | Shen et al. (2005) |
|  | 5’-GTA AAG AAG CCA GCC CAA CT-3’ |  |  |  |  | Wu et al. (2009) |
| AY161213 | 5’-CCT ACC TAT TTA CCT ACT TAC CTA CC-3’ | 4bp | 124-132 | 50 | HEX | Shen et al. (2005) |
|  | 5’-GAT GCT ATT AAG CAA CAG AC-3’ |  |  |  |  |  |
| AY217 | 5’-CTC ATG TGC TCA TTC ACT-3’ | 4bp | 108-116 | 48 | FAM | Shen et al. (2005) |
|  | 5’-TAG GGA GAA ACA GAT ACA T-3’ |  |  |  |  | Wu et al. (2009) |

**Table S2** Comparison of genetic diversity among the Qinling giant panda population and other populations from previous studies based on microsatellite markers.

| **Population** | **Sample size** | **Number of loci** | **MNA** | **H_E_** | **H_O_** | **References** |
| --- | --- | --- | --- | --- | --- | --- |
| **QIN** | 178 | 12 | 4.60 | 0.447 | 0.488 | Our study |
|  | 14 | 18 | 3.30 | -- | 0.57 | Lu et al.(2001) |
|  | 32 | 11 | 3.50 | 0.486 | 0.525 | Zhang et al.(2007) |
| **MS** | 29 | 11 | 4.80 | 0.559 | 0.561 | Zhang et al.(2007) |
| **QIO** | 40 | 11 | 5.30 | 0.61 | 0.595 | Zhang et al.(2007) |
| **LS** | 52 | 12 | 4.00 | 0.592 | 0.683 | Hu et al.(2010) |
| **XXL** | 41 | 18 | 4.56 | 0.656 | 0.704 | Zhu et al.(2011) |
| **DXL** | 42 | 18 | 4.67 | 0.643 | 0.66 | Zhu et al.(2011) |

*Landscape resistance hypotheses*

**Equation S1**: Landscape resistance as an Inverse Gaussian function of elevation:


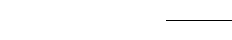


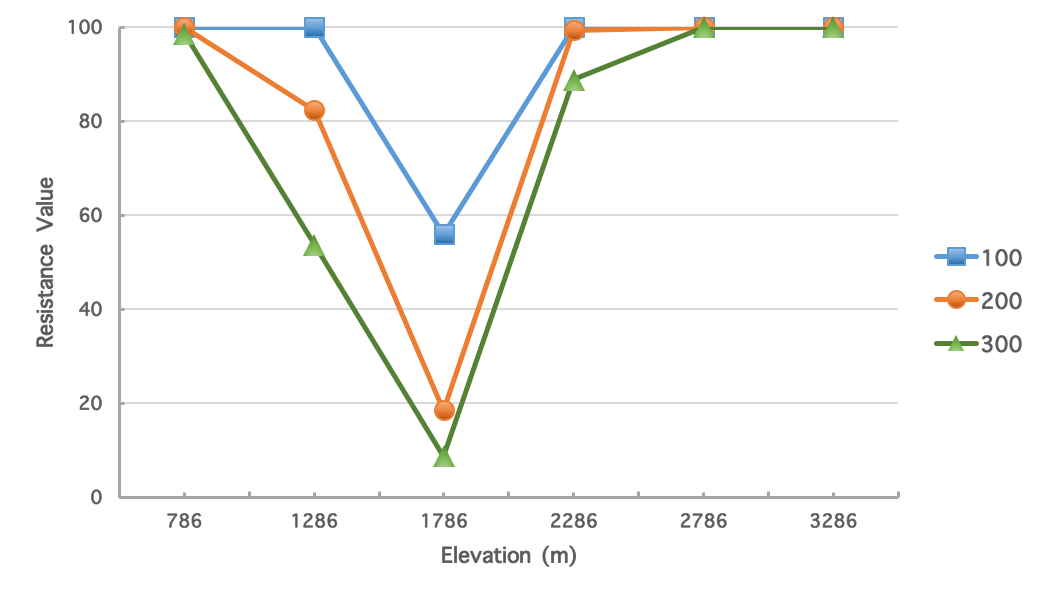


The figure above shows the resistance hypotheses with an optimal elevation of 2100 m and an R_max_ of 10. Series names are E_SD_ values. Higher values of E_SD_ represent lower contrast.

**Equation S2:** Landscape resistance as a modified heat load index function of slope aspect:


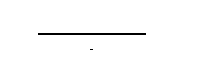


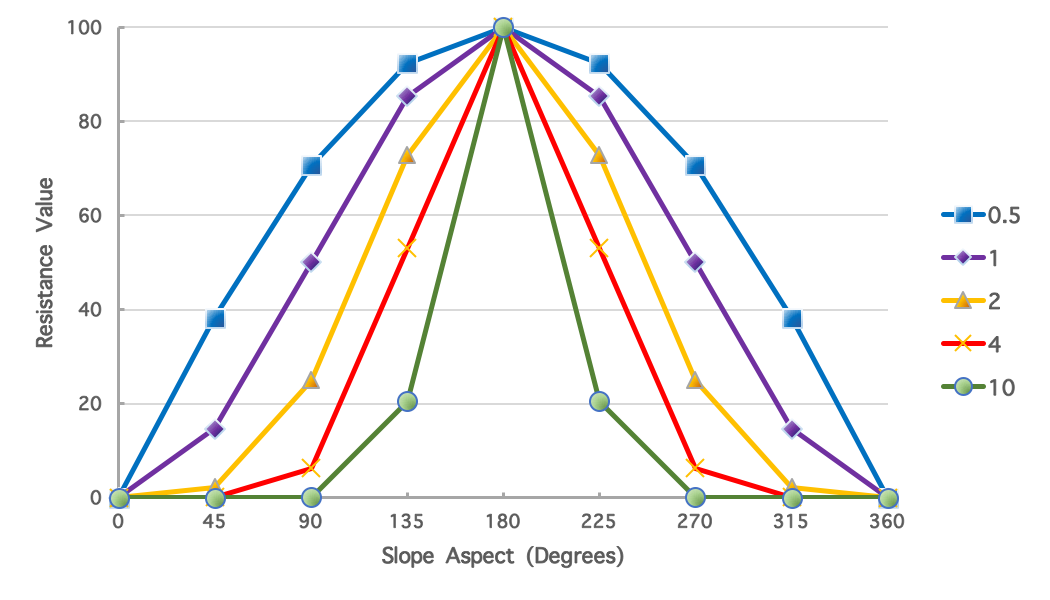


The above figure shows the resistance hypotheses with an optimal slope aspect of 0°and an R_max_ of 500. Series names represent different values of contrast (x). Higher values of x represent higher contrast.

**Equation S3:** Landscape resistance as a function of Topographic Complexity:


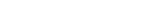


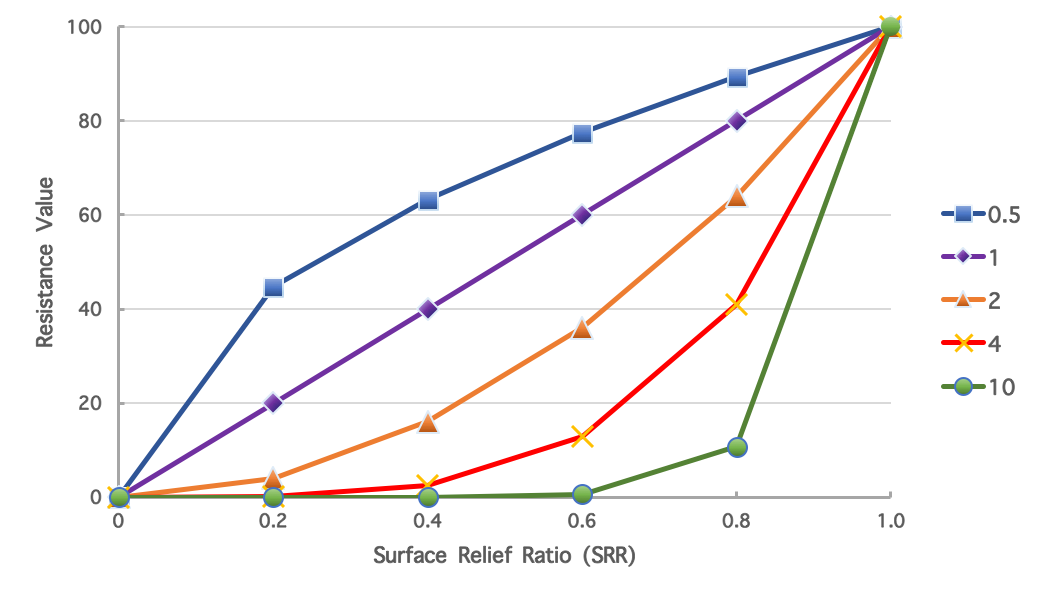


The above figure shows the resistance hypotheses for topographic complexity with R_max_ of 500. Series names represent different values of contrast (x).. Higher values of x represent higher contrast.

Raster maps of 30 x 30m in size sized for elevation, slope aspect, slope, DEM, river, vegetation, road and human disturbance (including farmlands and residences) were provided by the Forest Department of Shaanxi Province. We reclassified these landscape factors according to ecological hypotheses based on the biology of giant pandas and expert opinion. Hypotheses were tested using mathematical equations with the following parameters: *x* (contrast), and R_max_, (magnitude of the relationship). Different parameter values were tested to identified the best ecological and biological relationship between these landscape factors and gene flow. Parameter values were tested with both reciprocal causal modelling and mixed effect models.

**Elevation**: Elevation could be considered as a proxy for temperature as the temperature will decline when elevation increases. Elevation is also related to the available food resources which exist in a specific elevational range. We thus hypothesized there was an optimal elevation for giant panda movement. Elevation was divided into six elevation categories with an increment of 500 m. Landscape resistance was set as an Inverse Gaussian function (Equation S1) of elevation by varying the parameter values as follows: resistance maximum (R_max_) = 2, 10, 100, 500, 750, 1000; the optimal elevation (E_opt_) = the upper limit of each elevation group, and the standard deviation (E_SD_) =100, 200, 300 m.

**Slope aspect**: We hypothesized that slope aspect is related to the food availability and quality which could influence giant panda movement. McCune & Keon (2002) proposed an index of heat load (Equation S2) such that the northeastern aspect (45°) is the coolest and the southwestern aspect (225°) is the warmest in northern sphere. Aspect was divided into eight groups in 45° increments from 0° to 315°, while -1° (flat area) was recognized as a separate group. Landscape resistance was set as a function of aspect after optimization with the following parameters: R_max_ = 2, 10, 100, 500, 1000, the hypothesis optimal aspect (θ_opt_) = 0°, 45°, 90°, 135°, 180°, 225°, 270°, 315°, *x* = 0.5, 1, 2 ,4, 10 and flat areas were reclassified with R_max_/2.

**Slope**: Based on field observations, giant pandas prefer to use gentle slopes. Qinling giant pandas were inferred to use the slopes within the range from 0° to 30° (Pan *et al.* 1988). Thus, we hypothesized that there was an optimal slope gradient between 5° to 30° for giant panda movement. The slope gradient of the study area was classified into the following groups: 10°, 20°, 30°, 50°, 80°. Landscape resistance was set as the function of slope as an Inverse Gaussian function (the same similar as Equation S1) with the following parameters: R_max_ = 2, 10, 100, 500, 1000; the hypothesis optimal slope (θ_opt_) = 5°, 10°, 15°, 20°, 30°, 40° and standard deviation (θ_SD_) = 2°, 5°, 10°.

**Topographic complexity** **(TC)**: TC was tested based on the hypothesis that areas with higher TC values would increase the energy expenditure of movement for giant pandas. We reclassified the DEM using the Surface Relief Ratio (SRR) tool in ArcGIS v10.02 using 4 radii as the number of neighboring cells, which were 2, 10, 25 and 50, respectively. Equation S3 with the following parameters were used for this feature: R_max_ =2, 10, 100, 500, 1000; *x* = 0.5, 1, 2, 4, 10.

**Vegetation**: We sorted the vegetation of the study area into four major habitat groups: broadleaf forest, coniferous forest, broadleaf & coniferous mixed forest and other vegetation. According to the hypothesis that giant pandas prefer to inhabit broadleaf forest, coniferous forest, and coniferous mixed forest, a dichotomy model was constructed as follows: we assigned a value of “1” to the preferred forests habitats which represented the lowest resistance to giant panda movement. Twelve different R_max_ values (2, 5, 10, 50, 100, 250, 500, 750, 1000, 5000, 10000, 100000) were used to reclassify all the other vegetation types. In addition, six models were built as follows: an array of four resistance values (1, 444, 770, 1000) was produced by setting 1 and 1000 as the minimum (R_min_) and maximum (R_max_) respectively, when the two intermediate values were chosen at random following a linear distribution. We fixed the R_max_ to other type of vegetation while the other three values were randomly assigned to the preferred forest habitats.

**Rivers**: Only major rivers in the region were included in the analysis. No river areas were categorized with a value of “1”, represented as the lowest resistance to giant panda movement, while twelve different R_max_ values (2, 5, 10, 50, 100, 250, 500, 750, 1000, 5000, 10000, 100000) were assigned to rivers.

**Road**: Only the highways and other main roads in the region were included in the analysis and were tested if they could restrict giant panda movement. Considering that the obstructive effect of roads to giant panda movement might still exist in the areas adjacent to roads, we defined a buffer zone of 100 m on both sides of the road, and assigned it with the same landscape resistance value as for the roads. Resistance was modelled as follow: no road areas were set as a value of “1” which represented the lowest resistance to movement, while twelve different R_max_ values (2, 5, 10, 50, 100, 250, 500, 750, 1000, 5000, 10000, 100000) were assigned to roads and their buffer zones.

**Human disturbance**: This landscape factor consisted of two features, farmlands and residential areas. Similar to roads, a buffer zone of 1 500 m for residential area and a buffer zone of 300 m for farmland has been defined, respectively. The landscape resistance was modelled as follows: non-disturbed areas were categorized with a value of “1” which represented the lowest resistance to movement, while twelve different R_max_ values (2, 5, 10, 50, 100, 250, 500, 750, 1000, 5000, 10000, 100000) were assigned to disturbance elements and their buffer zones.

**Reference**

1. Castillo JA, Epps CW, Davis AR, Cushman SA (2014) Landscape effects on gene flow for a climate-sensitive montane species, the American pika. *Molecular Ecology* **23**, 843-856.
2. Lu Z, Johnson WE, Menotti-Raymond M, Yuhki N, Martenson JS, Mainka S, … O'Brien SJ (2001) Patterns of genetic diversity in remaining giant panda populations. *Conservation Biology* 15, 1596-1607
3. McCune B (2007) Improved estimates of incident radiation and heat load using non-parametric regression against topographic variables. *Journal of Vegetation Science* **18**, 751-754.
4. McCune B, Keon D (2002) Equations for potential annual direct incident radiation and heat load. *Journal of Vegetation Science* **13**, 603-606.
5. Russo IM, Sole CL, Barbato M, von Bramann U, Bruford MW (2016) Landscape determinants of fine-scale genetic structure of a small rodent in a heterogeneous landscape (Hluhluwe-iMfolozi Park, South Africa). *Scientific Reports* **6**.
6. Shen FJ, Watts P, Zhang ZH, Zhang AJ (2005) Enrichment of giant panda microsatellite markers using dynal magnet beads. *Yi Chuan Xue Bao*, **32**(5):457-462.
7. Wu H, Zhan XJ, Zhang ZJ, Zhu LF, Yan L, Li M, Wei FW (2009) Thirty-three microsatellite loci for noninvasive genetic studies of the giant panda (Ailuropoda melanoleuca). *Conservation Genetics* **10**, 649-652.
8. Evans JS, Oakleaf J, Cushman SA & Theobald D. An ArcGIS Toolbox for Surface Gradient and Geomorphometirc Modeling, version 2.0-0, 2014. Available: http://evansmurphy.wix.com/evansspatial (Accessed: 2 December 2014).
